# One-Center Location With Block and Euclidean Distance

**DOI:** 10.6028/jres.111.007

**Published:** 2006-04-01

**Authors:** P. M. Dearing, Phantipa Thipwiwatpotjana

**Affiliations:** Department of Mathematical Sciences, Clemson University, Clemson, SC 29634

**Keywords:** block distance, fundamental directions, linear program, one-center location problem, polar directions, polyhedral distance

## Abstract

A geometrical analysis is made of the dual simplex algorithm applied to a linear programming formulation of the one-center location problem in *IR*^2^ using block distance. A geometric rule is given, and shown to be equivalent to the minimum ratio rule of the simplex algorithm, for updating the dual basis. The geometric analysis is applied to the Euclidean distance one-center problem and yields an alternative updating procedure for the dual algorithm.

## 1. Introduction

The one-center location problem in the plane, also called the one facility min-max location problem, may be stated as follows: given *m* points ***p****_i_* ∈ *IR*^2^, *i* = 1, 2, …, *m*, and some distance function *d*(***x***, ***y***) for ***x***, ***y*** ∈ *IR*^2^, the problem is to determine the location of a point ***x*** ∈ *IR*^2^ that minimizes the maximum distance *d*(***p****_i_*, ***x***) over *i* = 1, …, *m*. The problem is denoted by P1 and written as follows:

$$P1:\begin{array}{cc} \underset{x\in IR^{2}}{\min} & \underset{i=1,\ldots m}{\max}d\left(p_{i},x\right) \end{array}.$$

The equivalent, constrained version of P1 is written as follows:

$$\begin{array}{l} \begin{array}{ccc} P1: & \min & z \end{array}\\ \;\begin{array}{cc} s.t. & z\geq d\left(p_{i},x\right) \end{array}\;i=1,\ldots ,m. \end{array}$$

Problem P1 was first reported by Sylvester [6] using Euclidean distance. A substantial number of papers have appeared on the one-center problem assuming a variety of distances. Articles and books with reviews of the literature include Hearn and Vijay, [4], Drezner [1], Drezner and Hamacher [2].

A related problem is the one-median, or total-cost, location problem in which the maximum operator is replaced by the summation operator. Both the center and the median problems have been studied extensively for Euclidean distance, for *l_p_* distances for 1 ≤ *p* ≤ ∞, and on networks [1,2]. Witzgall [9] considered the median problem for polyhedral norms and noted that the problem could be formulated as a linear programming problem. He also noted that “the linear program had special properties that should be exploited for an efficient solution”, and that “more research in this area is indicated.”

Ward and Wendell [8] considered both the one-median and the one-center problems using block distance, which is the special case of polyhedral distances in which the polytope is symmetric. For both the one-median and the one-center problems they reported two linear programming formulations based on characterizations of block distance in terms of fundamental directions and polar directions.

This paper considers the two linear programming formulations of the one-center problem with block distances, as presented by Ward and Wendell. The equivalence of these two formulations follows from the equivalence of the block distance representations. The dual simplex algorithm is applied to the linear programming formulation based on polar directions of the block distance, and a geometric interpretation is presented. This interpretation is applied to the Euclidean distance one-center problem and provides an alternative update procedure for the dual algorithm.

This paper actually considers a generalization of P1, the weighted one-center problem, in which there is a positive weight *w_i_* associated with each point ***p****_i_*, *i* = 1, …, *m*. The problem is denoted by P2 and the constrained version is stated as follows:

$$\begin{array}{l} \begin{array}{ccc} P2: & \min & z \end{array}\\ \;\begin{array}{cc} s.t. & z\geq w_{i}d\left(p_{i},x\right) \end{array}\;i=1,\ldots ,m. \end{array}$$

## 2. Block Distance

**Fig. 1:**
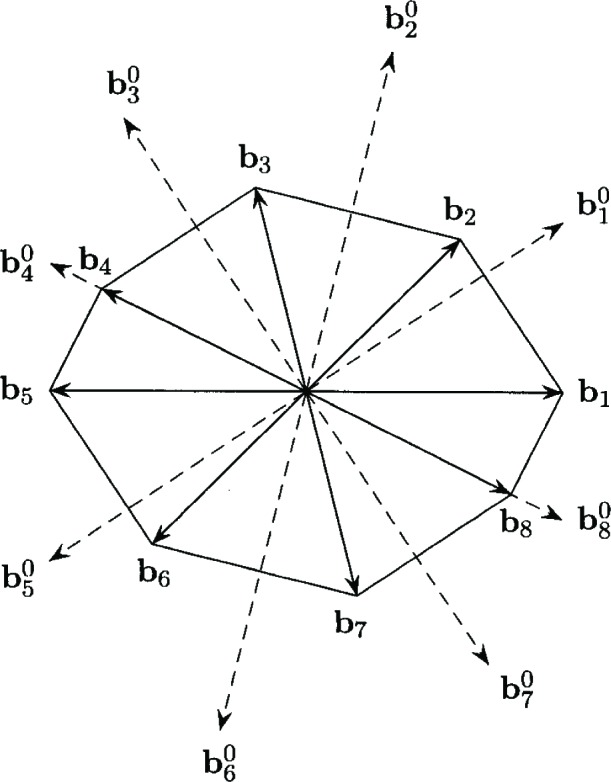
Fundamental directions *b_k_*, the unit ball, and polar directions $b_{k}^{0}$, *k* = 1, …, 8.

Block distance is a special case of general norms and were introduced to location problems by Witzgall [9] and by Ward and Wendell [7,8]. Block distance is defined in the plane with respect to a symmetric polytope as its unit ball, denoted by *B*. The polytope *B* is assumed to have 2*p* distinct extreme points, for some integer *p* ≥ 2. The vectors corresponding to the extreme points are called *fundamental directions*, and are denoted by ***b***_1_, ***b***_2_, …, ***b***_2_*_p_* where ***b****_p_*_+_*_k_* = −***b****_k_* for *k* = 1, …, *p*. Assume that the fundamental directions are ordered counter clockwise, and for notational convenience, let ***b***_2_*_p_*_+_*_k_* = ***b****_k_* for *k* = 1, …, *p*. Figure 1 shows an example with *p* = 4 fundamental directions and the corresponding unit ball.

The *block distance* between the points **x***_o_* and **x***_d_* with respect to a given set of 2*p* fundamental directions **b**_1_, …, ***b***_2_*_p_*, is denoted *d*_p_(***x****_o_*, ***x****_d_*) and is defined to be the objective function value of the following linear programming problem, denoted by LPD:

$$\begin{array}{l} \begin{array}{cc} \text{LPD}:d_{p}\left(x_{o},x_{d}\right)=\min & \sum_{k=1}^{2p}\alpha_{k} \end{array}\\ \;\begin{array}{cc} s.t. & \sum_{k=1}^{2p}b_{k}\alpha_{k} \end{array}=x_{o}-x_{d}\\ \begin{array}{cc} \;\alpha_{k}\geq 0 & k=1,\ldots ,2p. \end{array} \end{array}$$

For any two fundamental directions ***b****_j_*, ***b****_k_* ∈ *IR*^2^ with *j* ≠ *k* and *j* ≠ *k* + *p*, let Γ(***b****_j_*, ***b****_k_*) denote the cone in *IR*^2^ generated by ***b****_j_* and ***b****_k_*, that is, Γ(***b****_j_*, ***b****_k_*) = {*x* : *x* = ***b****_j_α_j_*+ ***b****_k_α_k_*, *α_j_*, *α_k_* ≥ 0}.

For any two points **x***_o_* and **x***_d_* ∈ *IR*^2^, the vector ***x****_o_* − ***x****_d_* must be in some cone generated by two adjacent fundamental directions, that is, ***x****_o_* − ***x****_d_* ∈ Γ(***b****_k_*, ***b****_k_*_+1_) for some *k* = 1, …, 2*p*. Thus ***x****_o_* − ***x****_d_* = ***b****_k_α_k_* + ***b****_k_*_+1_*α_k_*_+1_ for some non-negative scalars *α_k_* and *α_k_*_+1_. The vector ***x****_d_* − ***x****_o_* might also be in one or more cones generated by pairs of nonadjacent fundamental vectors. However, the following Property shows that an optimal basis to the linear program LPD must correspond to adjacent fundamental directions.

**Property** 1: Suppose ***b***_1_, ***b***_2_, ***b***_3_ ∈ *IR*^2^ are fundamental directions of some unit ball *B*. Suppose ***x*** ∈ Γ(***b***_1_, ***b***_2_) with ***x*** = ***b***_1_*α*_1_ + ***b***_2_*α*_2_, and *α*_1_, *α*_2_ ≥ 0. Suppose ***b***_3_ ∈ Γ(***b***_1_, ***b***_2_) with ***b***_3_ = ***b***_1_*β*_1_ + ***b***_2_*β*_2_, *β*_1_ + *β*_2_ > 1 and *β*_1_, *β*_2_ ≥ 0. If ***x*** ∈ Γ(***b***_1_, ***b***_3_) with ***x*** = ***b***_1_γ_1_ + ***b***_3_γ_3_, γ_1_, γ_3_ ≥ 0 then γ_1_ + γ_3_ < *α*_1_ + *α*_2_. If ***x*** ∈ Γ(***b***_3_, ***b***_2_) with ***x*** = ***b***_3_γ_3_ + ***b***_2_γ_2_, γ_3_, γ_2_ ≥ 0 then γ_3_ + γ_2_ < *α*_1_ + *α*_2_.

**Proof**: Suppose ***x*** ∈ Γ(***b***_1_, ***b***_3_), then ***x*** = ***b***_1_γ_1_ + ***b***_3_γ_3_ = ***b***_1_(*γ*_1_ + *γ*_3_*β*_1_) + ***b***_2_(*γ*_3_*β*_2_) by substitution. Since the representation of ***x*** as a nonnegative linear combination of ***b***_1_ and ***b***_2_ is unique, and ***x*** = ***b***_1_*α*_1_ + ***b***_2_*α*_2_ then *α*_1_ = *γ*_1_ + *γ*_3_*β*_1_ and *α*_2_ = *γ*_3_*β*_2_. Thus *α*_1_ + *α*_2_ = *γ*_1_ + *γ*_3_*β*_1_ + *γ*_3_*β*_2_ = *γ*_1_ + *γ*_3_(*β*_1_ + *β*_2_) > *γ*_1_ + *γ*_3_. If ***x*** ∈ Γ(***b***_1_, ***b***_2_) the proof is analogous.

Suppose that ***x****_o_* − ***x****_d_* ∈ Γ(***b****_k_*, ***b****_k_*_+1_) for some *k*. Then the 2 by 2 matrix $\begin{array}{cc} {}[b_{k} & b_{k+1}] \end{array}$.is a feasible basis for the linear program with

$$\left[\begin{array}{l} \alpha_{k}\\ \alpha_{k+1} \end{array}\right]={\left[\begin{array}{cc} b_{k} & b_{k+1} \end{array}\right]}^{-1}\left(x_{o}-x_{d}\right)$$

and

$$d\left(x_{o},x_{d}\right)=\alpha_{k}+\alpha_{k+1}=e^{T}{\left[\begin{array}{cc} b_{k} & b_{k+1} \end{array}\right]}^{-1}\left(x_{o}-x_{d}\right)$$

where *e^T^* = (1, 1).

Block distance may also be characterized in terms of the polar set *B*^0^ of the polytope *B*. The polar set *B*^0^ is also a symmetric polytope defined by $B^{0}=\left\{v:b_{k}^{T}v\leq 1,k=1,\ldots ,2p\right\}$. In general, the facets of *B* are in one-to-one correspondence with the extreme points of *B*^0^. In *IR*^2^, *B*^0^ has the same number of extreme points as *B*, which correspond to *polar directions* and are denoted by $b_{k}^{0}$.for *k* = 1, …, 2*p*. It may be shown that the polar directions are given by

$$b_{k}^{0T}=e^{T}{\left[\begin{array}{cc} b_{k} & b_{k+1} \end{array}\right]}^{-1},\text{for}\;k=1,\ldots ,2p.$$

Consider the dual of LPD, stated below as DLPD:

$$\begin{array}{l} \text{DLPD}:d_{p}\left(x_{o},x_{d}\right)=\max\;v^{T}\left(x_{o}-x_{d}\right)\\ \begin{array}{ccc} \;s.t. & b_{k}^{T}v & \leq \;1\;k=1,\ldots ,2p. \end{array} \end{array}$$

The constraint set of DLPD is the polar set *B*^0^ which, by the Representation Theorem [5], may be written as $B^{0}=\left\{v=\sum_{k=1}^{2p}b_{k}^{0}\lambda_{k},\sum_{k=1}^{2p}\lambda_{k}=1,\lambda_{k}\geq 0\right\}$. Substituting into the dual objective function gives the equivalent characterization of block distance in terms of polar directions:

$$d_{p}\left(x_{o},x_{d}\right)=\underset{k=1,\ldots ,2p}{\max}b_{k}^{0T}\left(x_{o}-x_{d}\right).$$

Block distances are used to model travel distance in which the directions of travel are restricted to the fundamental directions. The *l*_1_ distance is an example of a block distance with *p* = 2. Its fundamental directions are given by ***b***_1_ = ***ε***_1_, ***b***_2_ = ***ε***_2_, ***b***_3_ = −***ε***_1_, and ***b***_4_ = −***ε***_2_, where ***ε****_i_* is the *i*th unit vector in *IR*^2^. The polar directions for the *l*_1_ distance are given by $b_{1}^{0T}=\left(1,1\right)$, $b_{2}^{0T}=\left(-1,1\right)$, $b_{3}^{0}=-b_{1}^{0}$, and $b_{4}^{0}=-b_{2}^{0}$, which are also the fundamental directions of the *l*_∞_ distance, a block distance with *p* = 2.

Figure 1 illustrates a block distance with *p* = 4 and includes the fundamental directions ***b****_k_*, *k* = 1, …, 8, the unit ball and the polar directions $b_{k}^{0}$, *k* = 1, …, 8. The components of ***b****_k_* and $b_{k}^{0}$.used in Figure 1 are given below: $b_{1}^{T}=\left(1,0\right)$, $b_{2}^{T}=\left(\frac{3}{5},\frac{3}{5}\right)$, $b_{3}^{T}=\left(-\frac{1}{5},\frac{4}{5}\right)$, $b_{4}^{T}=\left(-\frac{4}{5},\frac{2}{5}\right)$, $b_{5}^{T}=\left(-1,0\right)$, $b_{6}^{T}=\left(-\frac{3}{5},-\frac{3}{5}\right)$, $b_{7}^{T}=\left(\frac{1}{5},-\frac{4}{5}\right)$, $b_{8}^{T}=\left(\frac{4}{5},-\frac{2}{5}\right)$.and $b_{1}^{0T}=\left(1,\frac{2}{3}\right)$, $b_{2}^{0T}=\left(\frac{1}{3},\frac{4}{3}\right)$, $b_{3}^{0T}=\left(-\frac{10}{14},\frac{15}{14}\right)$, $b_{4}^{0T}=\left(-1,\frac{1}{2}\right)$, $b_{5}^{0T}=\left(-1,-\frac{2}{3}\right)$, $b_{6}^{0T}=\left(-\frac{1}{3},-\frac{4}{3}\right)$, $b_{7}^{0T}=\left(\frac{10}{14},-\frac{15}{14}\right)$, $b_{8}^{0T}=\left(1,-\frac{1}{2}\right)$.

## 3. Linear Programming Formulations

Ward and Wendell [8] presented two linear programming formulations of the one facility minmax location problem (with all *w_i_* = 1) using block distance: one in terms of fundamental directions and one in terms of polar directions. These two formulations are given below for problem P2, and denoted as LP1 and LP2. The expression of block distance in terms of polar directions is substituted into problem P2 to obtain the following:

$$\begin{array}{l} \begin{array}{cc} \min & z \end{array}\\ \begin{array}{cc} s.t. & z\geq \max_{k=1,\ldots 2p} \end{array}w_{i}b_{k}^{0T}\left(p_{i}-x\right)\text{for}\;i=1,\ldots ,m. \end{array}$$

which is restated below as a linear program:

$$\begin{array}{l} \text{LP}1:\min\;z\\ \;s.t.\;z+w_{i}b_{k}^{0T}x\geq w_{i}b_{k}^{0T}p_{i}\;\text{for}\;i=1,\ldots ,m,\\ \;\text{and}\;k=1,\ldots 2p. \end{array}$$

Substituting the expression of block distance in terms of fundamental directions into problem P2 gives the following:

$$\begin{array}{l} \min\;z\\ \begin{array}{cc} s.t.\;z & \geq w_{i}\min\sum_{k=1}^{2p} \end{array}\alpha_{ik}\;\text{for}\;i=1,\ldots ,m\\ \begin{array}{cc} \;p_{i}-x=\sum_{k=1}^{2p}b_{k}\alpha_{ik} & \text{for} \end{array}\;i=1,\ldots ,m\\ \;\alpha_{ik}\;\geq 0\;\text{for}\;i=1,\ldots ,m,\;\text{and}\;k=1,\ldots ,2p \end{array}$$

which is restated as a linear program with all variables on the left as follows:

$$\begin{array}{l} \text{LP}2:\min\;z\\ \;s.t.\;z-w_{i}\sum_{k=1}^{2p}\alpha_{ik}\geq 0\;\text{for}\;i=1,\ldots ,m\\ \;x+\sum_{k=1}^{2p}b_{k}\alpha_{ik}=p_{i}\;\text{for}\;i=1,\ldots ,m\\ \;\alpha_{ik}\;\geq 0\;\text{for}\;i=1,\ldots ,m,\\ \;\text{and}\;k=1,\ldots ,2p \end{array}$$

The equivalence between the representations of block distance in terms of fundamental directions and in terms of polar directions implies that the formulations LP1 and LP2 are equivalent. Only problem LP1 will be considered in the subsequent development.

The dual to LP1 is given below where *π_i,k_*, *i* = 1, …, *m*, *k* = 1, …, 2*p*, are the dual variables.

$$\begin{array}{l} \max\sum_{i=1}^{m}\sum_{k=1}^{2p}\left(w_{i}b_{k}^{0T}p_{i}\right)\pi_{i,k}\\ s.t.\;\sum_{i=1}^{m}\sum_{k=1}^{2p}\pi_{i,k}\;=1\\ \;\sum_{i=1}^{m}\sum_{k=1}^{2p}w_{i}b_{k}^{0}\pi_{i,k}\;=0\\ \;\pi_{i,k}\;\geq 0\;i=1,\ldots ,m\;k=1,\ldots ,2p \end{array}$$

Since the dual constraints are of rank three, a dual basis has the form:

$$\left[\begin{array}{ccc} 1 & 1 & 1\\ w_{i_{1}}b_{k_{1}}^{0} & w_{i_{2}}b_{k_{2}}^{0} & w_{i_{3}}b_{k_{3}}^{0} \end{array}\right],$$

and the dual basic variables are denoted by $\pi_{i_{1},k_{1}}$, $\pi_{i_{2},k_{2}}$, $\pi_{i_{3},k_{3}}$, where *i_j_* ∈ {1, …, *m*} and *k_j_* ∈ {1, …, 2*p*} for *j* = 1, 2, 3. The three weighted polar directions that determine a dual feasible basis are called *basic weighted polar directions*.

Given a dual feasible basis, the dual simplex algorithm proceeds as follows. The basic weighted polar directions in the dual feasible basis correspond to active (equality) constraints in the primal, so that the variables z*, x* are determined by a solution to the following system of linear equations.

$$\left[\begin{array}{ll} 1 & w_{i_{1}}b_{k_{1}}^{0T}\\ 1 & w_{i_{2}}b_{k_{2}}^{0T}\\ 1 & w_{i_{3}}b_{k_{3}}^{0T} \end{array}\right]\;[\begin{array}{c} z*\\ x* \end{array}]=\left[\begin{array}{c} w_{i_{1}}b_{k_{1}}^{0T}p_{i_{1}}\\ w_{i_{2}}b_{k_{2}}^{0T}p_{i_{2}}\\ w_{i_{3}}b_{k_{3}}^{0T}p_{i_{3}} \end{array}\right].$$

If z*, x* are primal feasible, that is, if $z*\geq w_{i}b_{k}^{0T}\left(p_{i}-x*\right)$, for all *i* = 1, …, *m*, and *k* = 1, …, 2*p*, then x* and z* are optimal. Otherwise, for some point ***p***_q_ and some direction $b_{\tau }^{0}$,

$$z*<w_{q}b_{\tau }^{0T}\left(p_{q}-x*\right)$$

which implies that the point ***p****_q_* is outside the ball centered at x* with radius z*/*w_q_*. Choosing the most violated constraint corresponds to choosing a point of greatest weighted distance from x*.

The direction $b_{\tau }^{0}$.and the point ***p****_q_* determine the column $\left[\begin{array}{c} 1\\ w_{q}b_{\tau }^{0} \end{array}\right]$.that enters the dual basis. The leaving column may be determined by using the simplex rules, that is by using the following equations to compute the components *d*_1_, *d*_2_ and *d*_3_ of the direction vector corresponding to the basic columns:

$$\left[\begin{array}{c} 1\\ w_{i_{1}}b_{k_{1}}^{0} \end{array}\right]d_{1}+\left[\begin{array}{c} 1\\ w_{i_{2}}b_{k_{2}}^{0} \end{array}\right]d_{2}+\left[\begin{array}{c} 1\\ w_{i_{3}}b_{k_{3}}^{0} \end{array}\right]d_{3}=\left[\begin{array}{c} -1\\ -w_{q}b_{\tau }^{0} \end{array}\right].$$

Then the step size *α* and the leaving basic column are computed using the minimum ratio test:

$$\alpha =\underset{j=1,2,3}{\min}\left\{\frac{\pi_{i_{j},k_{j}}}{-d_{j}}:d_{j}<0\right\}=\frac{\pi_{i_{j*},k_{j*}}}{-d_{j*}}$$

and the column $\left[\begin{array}{c} 1\\ w_{i_{j*}}b_{k_{j*}}^{0} \end{array}\right]$.leaves the basis. The weighted polar directions in the new dual feasible basis are $w_{i_{j*-1}}b_{k_{j*-1}}^{0}$, and $w_{i_{j*+1}}b_{k_{j*+1}}^{0}$. The algorithm continues until primal feasibility is achieved. Problem LP1 is bounded and feasible, so that an optimal solution exists.

## 4. Geometric Interpretation of the Dual Basis Update

The update of the dual basis in the simplex algorithm applied to problem LP2 is analyzed in terms of the geometry associated with the basic weighted polar directions $w_{i_{j}}b_{k_{j}}^{0}$.

Consider a dual feasible basis

$$\left[\begin{array}{lll} 1 & 1 & 1\\ w_{i_{1}}b_{k_{1}}^{0} & w_{i_{2}}b_{k_{2}}^{0} & w_{i_{3}}b_{k_{3}}^{0} \end{array}\right]$$

with basic dual variables $\pi_{i_{1},k_{1}}$, $\pi_{i_{2},k_{2}}$, $\pi_{i_{3},k_{3}}$.for *i_j_* ∈ {1, …, *m*}, *k_j_* ∈ {1, …, 2*p*} and *j* = 1, 2, 3. Assume the weighted polar directions in the dual feasible basis are ordered counterclockwise with respect to *j* = 1, 2, 3. We adopt the notation that if *j* = 1, then *j* − 1 = 3, and if *j* = 3, then *j* + 1 = 1.

Geometrically, a dual feasible basis implies that the vector **0** may be expressed as a convex combination of basic weighted polar directions, that is,

$$\begin{array}{c} \begin{array}{ccc} \pi_{i_{1},k_{1}}+ & \pi_{i_{2},k_{2}}+ & \pi_{i_{3},k_{3}}=1 \end{array}\\ w_{i_{1}}b_{k_{1}}^{0}\pi_{i_{1},k_{1}}+w_{i_{2}}b_{k_{2}}^{0}\pi_{i_{2},k_{2}}+w_{i_{3}}b_{k_{3}}^{0}\pi_{i_{3},k_{3}}=0\\ \begin{array}{ccc} \pi_{i_{1},k_{1}}, & \pi_{i_{2},k_{2}}, & \pi_{i_{3},k_{3}},\geq 0. \end{array} \end{array}$$

If the dual feasible basis is non-degenerate, then $\pi_{i_{j},k_{j}}>0$.for *j* = 1, 2, 3 and **0** is a strict convex combination of the basic weighted polar directions. In this case each basic weighted polar direction is contained in the cone generated by the negative of the other two basic weighted polar directions, that is, $w_{i_{j-1}}b_{k_{j-1}}^{0}\;\in int\left(\Gamma \left(-w_{i_{j}}b_{k_{j}}^{0},-w_{i_{j+1}}b_{k_{j+1}}^{0}\right)\right)$.for each *j* = 1, 2, 3, where

$$\begin{array}{l} int\left(\Gamma \left(-w_{i_{j}}b_{k_{j}}^{0},-w_{i_{j+1}}b_{k_{j+1}}^{0}\right)\right)=\\ \left\{x:x=-w_{i_{j}}b_{k_{j}}^{0}\alpha -w_{k_{j+1}}b_{k_{j+1}}^{0}\beta ;\alpha ,\beta >0\right\}. \end{array}$$

**Fig. 2:**
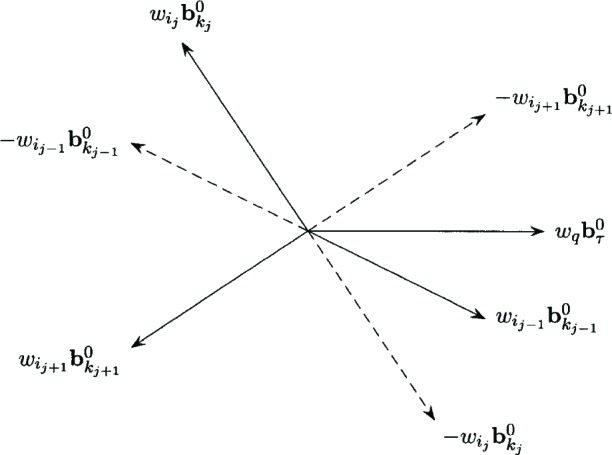
Non-degenerate basic feasible weighted polar directions.

Also, for any weighted polar direction $w_{q}b_{\tau }^{0}$, there is some *j* = 1, 2, 3 so that $w_{q}b_{\tau }^{0}\in \Gamma \left(-w_{i_{j}}b_{k_{j}}^{0},-w_{i_{j+1}}b_{k_{j+1}}^{0}\right)$. Figure 2 illustrates the non-degenerate case with solid arrows corresponding to the basic weighted polar directions $w_{i_{j}}b_{k_{j}}^{0}$.and dashed arrows corresponding to $-w_{i_{j}}b_{k_{j}}^{0}$.for *j* = 1, 2, 3. For the non-degenerate case the basic weighted polar directions form a simplex in *IR*^2^. Figure 2 also illustrates $w_{q}b_{\tau }^{0}\in \Gamma \left(-w_{i_{j}}b_{k_{j}}^{0},-w_{i_{j+1}}b_{k_{j+1}}^{0}\right)$.

**Fig. 3:**
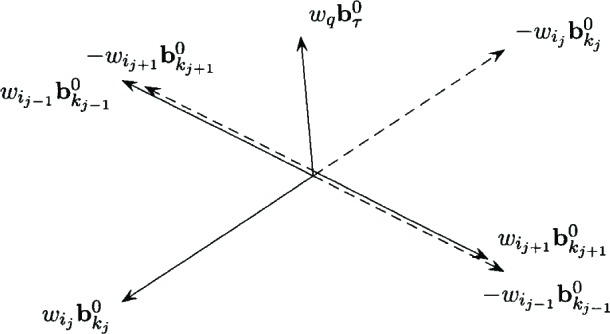
Degenerate basic feasible weighted polar directions.

In the degenerate case, $\pi_{i_{j},k_{j}}=0$.for exactly one *j* = 1, 2, or 3, and $\pi_{i_{j-1},k_{j-1}}>0$, $\pi_{i_{j+1},k_{j+1}}>0$. Figure 3 illustrates the degenerate case. Note that if $\pi_{i_{j},k_{j}}=0$, then $w_{i_{j+1}}b_{k_{j+1}}^{0}\in \Gamma \left(-w_{i_{j}}b_{k_{j}}^{0},-w_{i_{j-1}}b_{k_{j-1}}^{0}\right)$, and $w_{i_{j-1}}b_{k_{j-1}}^{0}\in \Gamma \left(-w_{i_{j}}b_{k_{j}}^{0},-w_{i_{j+1}}b_{k_{j+1}}^{0}\right)$, but $w_{i_{j}}b_{k_{j}}^{0}\notin \Gamma (-w_{i_{j+1}}b_{k_{j+1}}^{0},-w_{i_{j-1}}b_{k_{j-1}}^{0}$. Also, if $\pi_{i_{j},k_{j}}=0$, then for any weighted polar direction $w_{q}b_{\tau }^{0}$, $w_{q}b_{\tau }^{0}\in \Gamma \left(-w_{i_{j}}b_{k_{j}}^{0},-w_{i_{j+1}}b_{k_{j+1}}^{0}\right)$, or $w_{q}b_{\tau }^{0}\in \Gamma \left(-w_{i_{j}}b_{k_{j}}^{0},-w_{i_{j-1}}b_{k_{j-1}}^{0}\right)$, or $w_{q}b_{\tau }^{0}\in IR^{2}\backslash \left\{\Gamma \left(-w_{i_{j}}b_{k_{j}}^{0},-w_{i_{j+1}}b_{k_{j+1}}^{0}\right)\cup \Gamma \left(-w_{i_{j}}b_{k_{j}}^{0},-w_{i_{j-1}}b_{k_{j-1}}^{0}\right)\right\}$.

For the non-degenerate case, the basis update rule is given as follows: if $w_{q}b_{\tau }^{0}\in \Gamma \left(-w_{i}b_{k_{j}}^{0},-w_{i_{j+1}}b_{k_{j+1}}^{0}\right)$, for some *j*, and since $w_{i_{j-1}}b_{k_{j-1}}^{0}\in \Gamma \left(-w_{i_{j}}b_{k_{j}}^{0},-w_{i_{j+1}}b_{k_{j+1}}^{0}\right)$, then $w_{q}b_{\tau }^{0}$.replaces $w_{i_{j-1}}b_{k_{j-1}}^{0}$. The new basic weighted polar directions are $w_{q}b_{\tau }^{0}$, $w_{i_{j}}b_{k_{j}}^{0}$.and $w_{i_{j+1}}b_{k_{j+1}}^{0}$. If $w_{q}b_{\tau }^{0}\in int\left(\Gamma \left(-w_{i_{j}}b_{k_{j}}^{0},-w_{i_{j+1}}b_{k_{j+1}}^{0}\right)\right)$, then the new basic weighted polar directions are non-degenerate. If $w_{q}b_{\tau }^{0}$.coincides with $-w_{i_{j}}b_{k_{j}}^{0}$.or with $-w_{i_{j+1}}b_{k_{j+1}}^{0}$, then there is a tie for the replaced weighted polar direction and the new basis is degenerate. That is, if $w_{q}b_{\tau }^{0}$.coincides with $-w_{i_{j}}b_{k_{j}}^{0}$, then $w_{q}b_{\tau }^{0}\in \Gamma \left(-w_{i_{j}}b_{k_{j}}^{0},-w_{i_{j+1}}b_{k_{j+1}}^{0}\right)$.and $w_{q}b_{\tau }^{0}\in \Gamma \left(-w_{i_{j}}b_{k_{j}}^{0},-w_{i_{j-1}}b_{k_{j-1}}^{0}\right)$.and either $w_{i_{j-1}}b_{k_{j-1}}^{0}$.or $w_{i_{j+1}}b_{k_{j+1}}^{0}$.may be replaced. If $w_{q}b_{\tau }^{0}$.coincides with $-w_{i_{j+1}}b_{k_{j+1}}^{0}$, then $w_{q}b_{\tau }^{0}\in \Gamma \left(-w_{i_{j}}b_{k_{j}}^{0},-w_{i_{j+1}}b_{k_{j+1}}^{0}\right)$.and $w_{q}b_{\tau }^{0}\in \Gamma \left(-w_{i_{j+1}}b_{k_{j+1}}^{0},-w_{i_{j-1}}b_{k_{j-1}}^{0}\right)$.and either $w_{i_{j-1}}b_{k_{j-1}}^{0}$.or $w_{i_{j}}b_{k_{j}}^{0}$.may be replaced.

For the degenerate case suppose $\pi_{i_{j},k_{j}}=0$. If $w_{q}b_{\tau }^{0}\in \Gamma \left(-w_{i_{j}}b_{k_{j}}^{0},-w_{j_{j+1}}b_{k_{j+1}}^{0}\right)$.then $w_{q}b_{\tau }^{0}$.replaces $w_{i_{j-1}}b_{k_{j-1}}^{0}$. The new basic weighted polar directions are $w_{q}b_{\tau }^{0}$, $w_{i_{j}}b_{k_{j}}^{0}$.and $w_{i_{j+1}}b_{k_{j+1}}^{0}$. If $w_{q}b_{\tau }^{0}\in int\left(\Gamma \left(-w_{i_{j}}b_{k_{j}}^{0},-w_{i_{j+1}}b_{k_{j+1}}^{0}\right)\right)$.then the new basic weighted polar directions are non-degenerate. If $w_{q}b_{\tau }^{0}$.coincides with $-w_{i_{j}}b_{k_{j}}^{0}$, then $w_{q}b_{\tau }^{0}\in \Gamma \left(-w_{i_{j}}b_{k_{j}}^{0},-w_{i_{j+1}}b_{k_{j+1}}^{0}\right)$.and $w_{q}b_{\tau }^{0}\in \Gamma \left(-w_{i_{j}}b_{k_{j}}^{0},-w_{i_{j-1}}b_{k_{j-1}}^{0}\right)$.and either $w_{i_{j-1}}b_{k_{j-1}}^{0}$.or $w_{i_{j+1}}b_{k_{j+1}}^{0}$.may be replaced and the new basis is degenerate. However, if $w_{q}b_{\tau }^{0}$.coincides with $-w_{i_{j+1}}b_{k_{j+1}}^{0}$, then $w_{q}b_{\tau }^{0}\in \Gamma \left(-w_{i_{j}}b_{k_{j}}^{0},-w_{i_{j+1}}b_{k_{j+1}}^{0}\right)$.only, so that only $w_{i_{j-1}}b_{k_{j-1}}^{0}$.is replaced. The new basis is degenerate.

The degenerate case with $\pi_{i_{j},k_{j}}=0$.and $w_{q}b_{\tau }^{0}\in \Gamma \left(-w_{i_{j}}b_{k_{j}}^{0},-w_{i_{j-1}}b_{k_{j-1}}^{0}\right)$, is analogous to the degenerate case in the preceding paragraph with *j* + 1 interchanged with *j* − 1 throughout.

Finally, consider the degenerate case with $\pi_{i_{j},k_{j}}=0$.but $w_{q}b_{\tau }^{0}\notin \Gamma \left(-w_{i_{j}}b_{k_{j}}^{0},-w_{i_{j+1}}b_{k_{j+1}}^{0}\right)$, and $w_{q}b_{\tau }^{0}\notin \Gamma \left(-w_{i_{j}}b_{k_{j}}^{0},-w_{i_{j-1}}b_{k_{j-1}}^{0}\right)$. In this case $w_{q}b_{\tau }^{0}$.replaces $w_{i_{j}}b_{k_{j}}^{0}$. The new basic weighted polar directions are $w_{i_{j-1}}b_{k_{j-1}}^{0}$, $w_{q}b_{\tau }^{0}$.and $w_{i_{j+1}}b_{k_{j+1}}^{0}$.and remains degenerate.

The following Property shows that the new weighted polar directions determined by the geometric procedures above are basic feasible.

**Property 2**: Suppose the weighted polar directions $w_{i_{j-1}}b_{k_{j-1}}^{0}$, $w_{i_{j}}b_{k_{j}}^{0}$.and $w_{i_{j+1}}b_{k_{j+1}}^{0}$, are basic feasible. For either the non-degenerate or degenerate case, suppose $w_{q}b_{\tau }^{0}=-w_{i_{j}}b_{k_{j}}^{0}\beta_{j}-w_{i_{j+1}}b_{k_{j+1}}^{0}\beta_{j+1}$.with *β_j_*, *β_j_*_+1_ ≥ 0. Then the weighted polar directions $w_{i_{j+1}}b_{k_{j+1}}^{0}$, $w_{q}b_{\tau }^{0}$, and $w_{i_{j}}b_{k_{j}}^{0}$.are basic feasible.

**Proof:** The assumption $w_{q}b_{\tau }^{0}=-w_{i_{j}}b_{k_{j}}^{0}\beta_{j}-w_{i_{j+1}}b_{k_{j+1}}^{0}\beta_{j+1}$.with *β_j_*, *β_j_*_+1_ ≥ 0 implies $w_{i_{j}}b_{k_{j}}^{0}\beta_{j}+w_{q}b_{\tau }^{0}+w_{i_{j+1}}b_{k_{j+1}}^{0}\beta_{j+1}=0$. Let *r* = *β_j_* + 1 + *β_j_*_+1_ > 0. ${\pi^{\prime }}_{i_{j},k_{j}}=\frac{\beta_{j}}{r}\geq 0$, $\pi_{q,\tau }^{\prime }=\frac{1}{r}\geq 0$, ${\pi^{\prime }}_{i_{j}+1,k_{j}+1}=\frac{\beta_{j+1}}{r}\geq 0$. Thus ${\pi^{\prime }}_{i_{j},k_{j}}+\pi_{q,\tau }^{\prime }+{\pi^{\prime }}_{i_{j}+1,k_{j}+1}=1$, and the weighted polar directions $w_{i_{j+1}}b_{k_{j+1}}^{0}$, $w_{q}b_{\tau }^{0}$, and $w_{i_{j}}b_{k_{j}}^{0}$.are dual feasible. Observe that if either *β_j_* =0 or *β_j_*_+1_ = 0, the new basic weighted polar directions are degenerate.

The next property shows that the geometric replacement rule corresponds to the minimum ratio rule of the simplex algorithm.

**Property 3**: Suppose the weighted polar directions $w_{i_{j-1}}b_{k_{j-1}}^{0}$, $w_{i_{j}}b_{k_{j}}^{0}$, and $w_{i_{j+1}}b_{k_{j+1}}^{0}$, are basic feasible, and suppose $w_{q}b_{\tau }^{0}$.is the entering weighted polar direction and that $w_{q}b_{\tau }^{0}\in \Gamma \left(-w_{i_{j}}b_{k_{j}}^{0},-w_{i_{j+1}}b_{k_{j+1}}^{0}\right)$. The geometric rule that $w_{q}b_{\tau }^{0}$.replaces $w_{i_{j-1}}b_{k_{j-1}}^{0}$.is equivalent to the minimum ratio rule of the simplex algorithm.

**Proof:** The proof is given for the non-degenerate case. The proof of the degenerate case is similar. First we show that if $w_{q}b_{\tau }^{0}\in \Gamma \left(-w_{i_{j}}b_{k_{j}}^{0},-w_{i_{j+1}}b_{k_{j+1}}^{0}\right)$, then $\min_{j=1,2,3}\left\{\frac{\pi_{i_{j},k_{j}}}{-d_{j}}:d_{j}<0\right\}=\frac{\pi_{i_{j-1},k_{j-1}}}{-d_{j-1}}$.

Dual feasibility implies the following equations:

$$w_{i_{j-1}}b_{k_{j-1}}^{0}\pi_{i_{j-1},k_{j-1}}+w_{i_{j}}b_{k_{j}}^{0}\pi_{i_{j},k_{j}}+w_{i_{j+1}}b_{k_{j+1}}^{0}\pi_{i_{j+1},k_{j+1}}=0$$ (1)

$$\begin{array}{ccc} \pi_{i_{j-1},k_{j-1}}+ & \pi_{i_{j},k_{j}}+ & \pi_{i_{j+1},k_{j+1}}=1. \end{array}$$ (2)

The components of the direction vector determined by the vector $w_{q}b_{\tau }^{0}$.are given by the following equations:

$$-w_{i_{j-1}}b_{k_{j-1}}^{0}d_{j-1}-w_{i_{j}}b_{k_{j}}^{0}d_{j}-w_{i_{j+1}}b_{k_{j+1}}^{0}d_{j+1}=w_{q}b_{\tau }^{0}$$ (3)

$$\begin{array}{ccc} d_{j-1}- & d_{j}- & d_{j+1} \end{array}=1.$$ (4)

The assumption that $w_{q}b_{\tau }^{0}\in \Gamma \left(-w_{i_{j}}b_{k_{j}}^{0},-w_{i_{j+1}}b_{k_{j+1}}^{0}\right)$.implies

$$-w_{i_{j}}b_{k_{j}}^{0}\beta_{i_{j},k_{j}}-w_{i_{j+1}}b_{k_{j+1}}^{0}\beta_{i_{j+1},k_{j+1}}=w_{q}b_{\tau }^{0},$$ (5)

where $\beta_{i_{j},k_{j}}\geq 0$.and $\beta_{i_{j+1},k_{j+1}}\geq 0$. Equations (3) and (5) combine to give

$$w_{i_{j-1}}b_{k_{j-1}}^{0}\left(-d_{j-1}\right)+w_{i_{j}}b_{k_{j}}^{0}\left(\beta_{i_{j},k_{j}}-d_{j}\right)+w_{i_{j+1}}b_{k_{j+1}}^{0}\left(\beta_{i_{j+1},k_{j+1}}-d_{j+1}\right)=0$$

and equation (4) implies that

$$-d_{j-1}+\left(\beta_{i_{j+1},k_{j+1}}-d_{j}\right)+\left(\beta_{i_{j+1},k_{j+1}}-d_{j+1}\right)=\beta_{i_{j},k_{j}}+\beta_{i_{j+1},k_{j+1}}+1.$$

Dividing through the last equation by the right hand side, and comparing the resulting two equations to (1) and (2), shows that

$$\pi_{i_{j-1},k_{j-1}}=\frac{-d_{j-1}}{\beta_{i_{j},k_{j}}+\beta_{i_{j+1},k_{j+1}}+1}$$

so that $d_{i_{j-1}}<0$.since $\pi_{i_{j-1},k_{j-1}}>0$. Equation (1) implies that

$$-w_{i_{j-1}}b_{k_{j-1}}^{0}=w_{i_{j}}b_{k_{j}}^{0}\frac{\pi_{i_{j},k_{j}}}{\pi_{i_{j-1},k_{j-1}}}+w_{i_{j+1}}b_{k_{j+1}}^{0}\frac{\pi_{i_{j+1},k_{j+1}}}{\pi_{i_{j-1},k_{j-1}}}.$$

Substitute this expression into equation (3), multiply through by $\pi_{i_{j-1},k_{j-1}}$.and divide through by −*d_j_*_−1_ to get

$$\begin{array}{l} \left(\pi_{i_{j},k_{j}}+\frac{\pi_{i_{j-1},k_{j-1}}}{-d_{j-1}}d_{j}\right)w_{i_{j}}b_{k_{j}}^{0}+\left(\pi_{i_{j+1},k_{j+1}}+\frac{\pi_{i_{j-1},k_{j-1}}}{-d_{j-1}}d_{j+1}\right)w_{i_{j+1}}b_{k_{j+1}}^{0}\\ +\frac{\pi_{i_{j-1},k_{j-1}}}{-d_{j-1}}w_{q}b_{\tau }^{0}=0. \end{array}$$ (6)

If *d_j_* ≥ 0, the first coefficient is positive. If *d_j_* < 0, the first coefficient is non-negative if and only if

$$\frac{\pi_{i_{j-1},k_{j-1}}}{-d_{j-1}}\leq \frac{\pi_{i_{j},k_{j}}}{-d_{j}}.$$

A similar argument holds for the second coefficient. The third coefficient is positive. Thus, the new coefficients are non-negative if and only if $\alpha =\frac{\pi_{i_{j-1},k_{j-1}}}{-d_{j-1}}$.is the minimum ratio.

To prove the converse, suppose that $\min_{j=1,2,3}\left\{\frac{\pi_{i_{j},k_{j}}}{-d_{j}}:d_{j}<0\right\}=\frac{\pi_{i_{j-1},k_{j-1}}}{-d_{j-1}}$, and show that $w_{q}b_{\tau }^{0}\in \Gamma \left(-w_{i_{j}}b_{k_{j}}^{0},-w_{i_{j+1}}b_{k_{j+1}}^{0}\right)$.

By the minimum ratio assumption, *d_j_*_−1_ < 0. If *d_j_* < 0, then $\frac{\pi_{i_{j-1},k_{j-1}}}{-d_{j-1}}\leq \frac{\pi_{i_{j},k_{j}}}{-d_{j}}$.by the minimum ratio, and the first coefficient in equation (6) is non-negative. If *d_j_* ≥ 0, the first coefficient in equation (6) is non-negative. A similar argument holds for *d_j_*_+1_ and the second coefficient in equation (6). Thus $w_{q}b_{\tau }^{0}\in \Gamma \left(-w_{i_{j}}b_{k_{j}}^{0},-w_{i_{j+1}}b_{k_{j+1}}^{0}\right)$.□

Properties 2 and 3 provide a geometric rule that could be used to determine the leaving column of a dual basis in the basis update step. However, the equivalent minimum ratio rule of the simplex algorithm is more efficient.

In the next section, the equivalence is used to show that the minimum ratio rule may be used to update the dual algorithm applied to the Euclidean distance one-center problem, which is an improvement over existing geometrical update rules.

The results of the last two sections may be extended to polyhedral norms with consideration given to the asymmetry. The preceding results may also be extended to *IR^n^* with additional complexities and notation.

## 5. Euclidean Distance Problem

Considered next is the one-center location problem using Euclidean distance and assuming all weights *w_i_* = 1. The constrained version of this problem is written as follows:

$$\begin{array}{l} P2:\min\;z\\ \;s.t.\;z\geq l_{2}\left(p_{i},x\right)i=1,\ldots ,m. \end{array}$$

Euclidean distance may be considered as a block distance with *p* → ∞ and whose unit ball is the circle of radius 1. There is a fundamental direction for every point on the unit circle and each fundamental direction is of unit length. The polar directions for Euclidean distance are identical to the fundamental directions. Given two points ***p****_i_* and ***x***, the unit vector $\frac{p_{i}-x}{\left|p_{i}-x\right|}$.represents the polar direction from ***x*** to ***p****_i_*. The polar direction that maximizes the dot product with ***p****_i_* − ***x*** is the unit vector $\frac{p_{i}-x}{\left|p_{i}-x\right|}$. Thus the Euclidean distance may be interpreted in terms of polar directions and is written as: $l_{2}\left(p_{i},x\right)=\frac{p_{i-x}}{\left|p_{i-x}\right|}\left(p_{i},-x\right)$. Problem P2 is then written as:

$$\begin{array}{l} P2:\min\;z\\ \;s.t.\;z\geq \frac{p_{i}-x}{\left|p_{i}-x\right|}\left(p_{i}-x\right)i=1,\ldots ,m. \end{array}$$

An alternative and equivalent statement of problem P2 is to use the squared Euclidean distance, that is, $l_{2}^{2}\left(p_{i},x\right)={\left(p_{i}-x\right)}^{T}\left(p_{i}-x\right)$. The squared Euclidean distance between the points ***p****_i_* and ***x*** may also be considered as a block distance with *p* → ∞ and whose unit ball is the circle of radius |***p****_i_* − ***x***|. There is a fundamental direction for every point on the unit circle, and the polar directions are identical to the fundamental directions. Given two points ***p****_i_* and ***x***, ***p****_i_* − ***x*** is the polar direction from ***x*** to ***p****_i_*. Problem P2 for the squared Euclidean distance is written as:

$$\begin{array}{l} P2:\min\;z\\ \;s.t.\;z\geq {\left(p_{i}-x\right)}^{T}\left(p_{i}-x\right)i=1,\ldots ,m. \end{array}$$

The squared Euclidean distance problem has the advantage that the constraints are differentiable. This leads to the Karush-Kuhn-Tucker conditions for problem P2: A point ***x*** and a radius *z* are optimal to P2 if and only if there exists π*_i_* for *i* = 1, …, *n*, such that:

$$z\geq {\left(p_{i}-x\right)}^{T}\left(p_{i}-x\right)\;i=1,\;\ldots ,\;m$$ (7)

$$\sum_{i=1}^{n}\pi_{i}=1$$ (8)

$$\sum_{i=1}^{n}\left(p_{i}-x\right)\pi_{i}=0$$ (9)

$$\pi_{i}\geq 0\;i=1,\;\ldots ,\;m$$ (10)

$$\left(z-{\left(p_{i}-x\right)}^{T}\left(p_{i}-x\right)\right)\pi_{i}=0\;i=1,\;\ldots ,\;m$$ (11)

Conditions (7) represent primal feasibility, conditions (8), (9), (10) represent dual feasibility, and conditions (11) represent complementary slackness. The dual feasibility conditions are analogous to constraints of the dual problem of LP1. For a given ***x***, the dual constraints are of rank three and a dual basis has the form:

$$\left[\begin{array}{ccc} 1 & 1 & 1\\ p_{i_{1}}-x & p_{i_{2}}-x & p_{i_{3}}-x \end{array}\right].$$

The dual variables are denoted by $\pi_{i_{1}}$, $\pi_{i_{2}}$.and $\pi_{i_{3}}$.respectively. The polar directions $p_{i_{j}}-x$.for *j* = 1, 2, 3 that determine a dual feasible basis are called basic polar directions.

Elzinga and Hearn [3], report an efficient dual based algorithm for solving the Euclidean distance min-max problem that is described as follows. Choose three points $p_{i_{j}},i_{j}\in \left\{1,\ldots ,m\right\}$.and *j* = 1, 2, 3. In the primal phase of the algorithm, the min-max solution ***x*** for these three points is determined as follows. If the three points form an acute triangle, then ***x*** is the intersection of the perpendicular bisectors of the line segment between any two pairs of points. If the three points form an obtuse triangle, then ***x*** is the mid point of the line segment between the pair of points forming the longest side (opposite the obtuse angle) of the triangle. In either case, *z* is the maximum distance from ***x*** to the three points.

Next the solution ***x*** and *z* is checked for primal feasibility. If the distance between ***x*** and ***p****_i_* does not exceed *z* for all *i* = 1, …, *m*, then ***x*** and *z* is an optimal solution. Otherwise, some point ***p****_τ_* is chosen that violates primal feasibility. The Elzinga-Hearn algorithm provides a geometric procedure to determine which of the three points $p_{i_{j}}$, *j* = 1, 2, 3 is replaced by ***p****_τ_* and the algorithm continues.

**Fig. 4:**
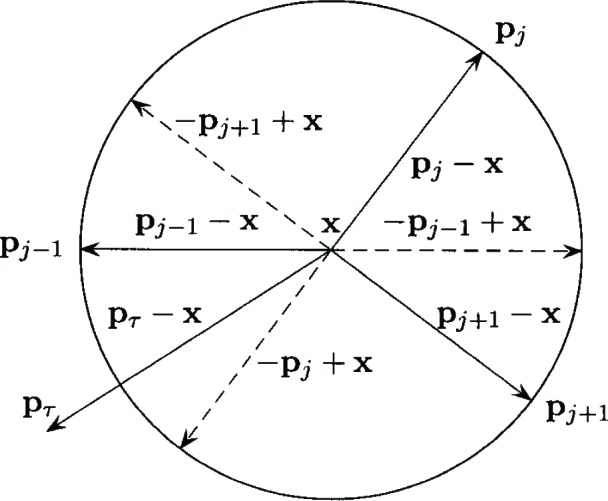
Euclidean distance example.

The Elzinga-Hearn geometric procedure for determining which point is replaced by ***p****_τ_* is described as follows: Determine the point $p_{i_{j}}$, *j* = 1, 2, 3 that is of greatest distance from ***p****_τ_*, say $p_{i_{j}}$. If the point $p_{i_{j-1}}$.(or $p_{i_{j+1}}$) and ***p****_τ_* are on the same side of the line through $p_{i_{j}}$.and ***x***, then ***p****_τ_* replaces $p_{i_{j-1}}$.(or $p_{i_{j+1}}$). Figure 4 illustrates an example where ***p****_τ_* replaces $p_{i_{j-1}}$.

In terms of dual feasibility, the three points $p_{i_{j}}$.for *j* = 1, 2, 3 correspond to the basic polar directions $p_{i_{j}}-x$.in the dual basis. If the three points form an acute triangle, the corresponding basic polar directions are non-degenerate and form a simplex in *IR*^2^. If the three points form an obtuse triangle, the corresponding basic polar directions are degenerate. The point ***p****_τ_* corresponds to the polar direction ***p****_τ_* − ***x*** that will become basic and replace some existing basic polar direction. Figure 4 illustrates the basic polar directions.

The Elzinga-Hearn procedure is now compared to the geometric replacement rule developed above. Suppose $p_{\tau }-x\in \Gamma \left(-p_{i_{j}}+x,-p_{i_{j+1}}+x\right)$. Then the point farthest from ***p****_τ_* is either $p_{i_{j}}$.or $p_{i_{j+1}}$. By dual feasibility, $p_{i_{j-1}}-x\in \Gamma \left(-p_{i_{j}}+x,-p_{i_{j+1}}+x\right)$. The replacement rule developed above states that ***p****_τ_* − ***x*** replaces. The inclusion $p_{i_{j-1}}-x\in \Gamma \left(-p_{i_{j}}+x,-p_{i_{j+1}}+x\right)$.implies that $p_{i_{j-1}}$.and ***p****_τ_* are on the same side of the line through $p_{i_{j}}$.and ***x***, and that $p_{i_{j-1}}$.and ***p****_τ_* are on the same side of the line through $p_{i_{j+1}}$.and ***x***. Thus, the replacement rules are equivalent. If the basic polar directions are degenerate, a similar argument shows the equivalence of the replacement rules.

Since the geometric replacement rule for block distance is equivalent to the minimum ratio rule of the basis update, the minimum ratio rule provides an alternative to the Elzinga-Hearn geometric rule for selecting the point to be replaced in the dual based algorithm for the Euclidean distance min-max location problem.

The algebraic replacement rule for the un-weighted Euclidean distance min-max problem has been extended to the weighted Euclidean distance min-max problem in a subsequent paper by the first author.
